# Intravenously transplanted mesenchymal stromal cells: a new endocrine reservoir for cardioprotection

**DOI:** 10.1186/s13287-022-02922-z

**Published:** 2022-06-17

**Authors:** Anan Huang, Yue Liu, Xin Qi, Shang Chen, Haoyan Huang, Jun Zhang, Zhibo Han, Zhong-Chao Han, Zongjin Li

**Affiliations:** 1 Nankai University School of Medicine, 94 Weijin Road, Tianjin, 300071 China; 2 Department of Cardiology, Tianjin Union Medical Center, 190 Jieyuan Road, Tianjin, 300121 China; 3 The Key Laboratory of Bioactive Materials, Ministry of Education, The College of Life Sciences, Nankai University, Tianjin, China; 4 Department of Pain Medicine, Tianjin Union Medical Center, Nankai University, Tianjin, China; 5 Jiangxi Engineering Research Center for Stem Cell, Shangrao, Jiangxi China; 6 Tianjin Key Laboratory of Engineering Technologies for Cell Pharmaceutical, National Engineering Research Center of Cell Products, AmCellGene Co., Ltd, Tianjin, China; 7 Henan Key Laboratory of Medical Tissue Regeneration, Xinxiang Medical University, Xinxiang, China

**Keywords:** Intravenous infusion, Mesenchymal stromal cells, Molecular imaging, Extracellular vesicles, Doxorubicin, Cardiotoxicity, Endoplasmic reticulum stress, miRNA

## Abstract

**Background:**

Intravenous administration of mesenchymal stromal cells (MSCs) has an acknowledged competence of cardiac repair, despite a lack of systematic description of the underlying biological mechanisms. The lung, but not the heart, is the main trapped site for intravenously transplanted MSCs, which leaves a spatial gap between intravenously transplanted MSCs and the injured myocardium. How lung-trapped MSCs after intravenous transplantation rejuvenate the injured myocardium remains unknown.

**Methods:**

MSCs were isolated from human placenta tissue, and DF-MSCs or Gluc-MSCs were generated by transduced with firefly luciferase (Fluc)/enhanced green fluorescence protein (eGFP) or Gaussia luciferase (Gluc) lactadherin fusion protein. The therapeutic efficiency of intravenously transplanted MSCs was investigated in a murine model of doxorubicin (Dox)-induced cardiotoxicity. Trans-organ communication from the lung to the heart with the delivery of blood was investigated by testing the release of MSC-derived extracellular vesicles (MSC-EVs), and the potential miRNA inner MSC-EVs were screened out and verified. The potential therapeutic miRNA inner MSC-EVs were then upregulated or downregulated to assess the further therapeutic efficiency

**Results:**

Dox-induced cardiotoxicity, characterized by cardiac atrophy, left ventricular dysfunction, and injured myocardium, was alleviated by consecutive doses of MSCs. These cardioprotective effects might be attributed to suppressing GRP78 triggering endoplasmic reticulum (ER) stress-induced apoptosis in cardiomyocytes. Our results confirmed that miR-181a-5p from MSCs-derived EVs (MSC-EVs) inhibited GRP78. Intravenous DF-MSCs were trapped in lung vasculature, secreted a certain number of EVs into serum, which could be confirmed by the detection of eGFP^+^ EVs. GLuc activity was increased in serum EVs from mice administrated with GLuc-MSCs. MiR-181a-5p, inhibiting GRP78 with high efficacy, was highly expressed in serum EVs and myocardium after injecting consecutive doses of MSCs into mice treated with Dox. Finally, upregulation or downregulation of miR-181a-5p levels in MSC-EVs enhanced or weakened therapeutic effects on Dox-induced cardiotoxicity through modulating ER stress-induced apoptosis.

**Conclusions:**

This study identifies intravenously transplanted MSCs, as an endocrine reservoir, to secrete cardioprotective EVs into blood continuously and gradually to confer the trans-organ communication that relieves Dox-induced cardiotoxicity.

**Supplementary Information:**

The online version contains supplementary material available at 10.1186/s13287-022-02922-z.

## Introduction

Intravenous infusion of mesenchymal stromal cells (MSCs) is a prominent clinical therapeutic strategy in multiple diseases, even coronavirus disease 2019 (COVID-19) [1–3]. Unlike other delivery routes, such as intracoronary and intramyocardial infusion, intravenous injection has the prominent superiority of repeated, noninvasive, and easy operation. The lung, but not the heart, is the main trapped site for intravenously injected MSCs. Therefore, how intravenously infused MSCs bridge the spatial gap between the lung and heart to achieve cardiac repair remains a challenge [4, 5].

Placenta-derived mesenchymal stromal cells (MSCs) are isolated from the fetal placental tissue including four major regions of amniotic epithelial, amniotic mesenchymal, chorionic mesenchymal, and chorionic trophoblastic [6, 7]. Normally, placenta-derived MSCs can be cultured with the characteristics of plastic-adherent and have a tendency to form fibroblast colony-forming units [8]. Like other types of mesenchymal stromal cells, a specific pattern of surface antigen markers can be tested to identify placenta-derived MSCs with over 95% positive expression of CD90, CD73, and CD105 and below 2% negative expression of CD45, CD34, CD14, and HLA-DR [6]. In addition, the differentiation potential of placenta-derived MSCs can be toward three lineages including osteogenic, adipogenic, and chondrogenic [9, 10].

Emerging evidence supports trans-organ communication among endogenous cells from diverse organs plays a critical role in tissue repair or regeneration. During exercise, skeletal myocytes or vascular endothelial cells secrete certain factors into the blood to mediate trans-organ communication [11–13]. Current findings inspire us to investigate whether intravenously infused MSCs can be a new endocrine reservoir, which communicates with cells from distal organs [14, 15]. Extracellular vesicles (EVs) secreted by cells or tissue, known as an evolutionarily conserved process, can encapsulate abundant signal molecules and transfer them to distant recipient cells, minimizing the destruction of certain blood components [14, 16–18]. Accumulated evidence reveals multiple molecular imaging is prone to the trace of transplanted MSCs and even MSC-EVs [14, 16, 19]. Previous literature reported that intravenous EVs transplantation can be cleared from blood promptly and are undetectable in blood after a few hours [20]. Compared to that, intravenously infused MSCs are capable of releasing EVs into blood continuously and gradually [16, 21]. In addition, EVs derived from intravenously administrated MSCs can be detected in peripheral blood which suggests that intravenously transplanted MSCs are capable of releasing EVs in vivo [22]. MiRNAs, as a critical part of noncoding RNA loaded in EVs, have a negative regulating effect on protein expression. In stem cell therapy, miRNA encapsulated in MSC-EVs has been highlighted as one of the promising treatments in tissue repair and anti-senescence [16, 23]. Meanwhile, miRNA may have a better degradation-free delivery efficiency once encapsulated into EVs [24]. However, it remains unclear whether intravenously transplanted MSCs released EVs into the blood and then delivered into the injured myocardium and the miRNAs loaded in MSC-EVs participate in the cardiac repair.

Chemotherapy agent is a two-edged sword with outstanding clinical benefits and uncontrolled side effects [25]. Of these, cardiotoxicity occupies the highest frequency as the leading cause of morbidity and mortality in cancer survivors [25–27]. Doxorubicin (Dox), as a classic chemotherapy agent, has been well investigated due to the increasing cases of Dox-induced cardiotoxicity [28]. Dox-induced cardiotoxicity always is characterized by a series of morphological changes including cardiac atrophy, vacuolation of cardiomyocytes, and dilated ER [29–31]. Current treatment against cardiotoxicity does not reduce morbidity and mortality in cancer survivors. Therefore, novel treatment is urgently needed for those vulnerable cancer patients. Although MSCs are a type of stem cell, clinical trials and basic scientific studies do not observe any acceleration of tumor progress as intravenous MSCs administered, which supports the potential use of MSCs in treating Dox-induced cardiotoxicity through trans-organ communication [32–34].

To delineate the biological mechanism of how intravenous MSCs transplantation bridge the gap between the lung and heart in cardiac repair, we built a murine model of Dox-induced cardiotoxicity and transplanted MSCs are injected into mice intravenously. Multiple engineering MSCs were generated to facilitate the track of MSCs and MSC-EVs in vivo. Here, we hypothesized that intravenously transplanted MSCs, trapped in the pulmonary vasculature, secreted specific miRNA enriched EVs into the injured myocardium through peripheral blood. In this study, miR-181a-5p was identified as a potential candidate in MSC-EVs, which downregulated the signaling pathway of glucose-regulated protein 78 (GRP78) triggered endoplasmic reticulum (ER) stress. Upregulation or downregulation of miR-181a-5p levels in MSC-EVs intensified or weakened the therapeutic effects on Dox-induced cardiotoxicity.

## Materials and methods

### Cell preparation

The collection of human placenta-derived mesenchymal stromal cells (MSCs) was in line with previous literature [35]. MSCs with a double-fusion reporter gene (DF-MSCs) were transduced with a lentiviral vector carrying a human ubiquitin promoter driving firefly luciferase (*Fluc*) and enhanced green fluorescence protein (eGFP) as previously described [35, 36]. Gluc-MSCs expressing lactadherin fusion proteins with *Gaussia* luciferase (Gluc) were constructed as previously reported [16]. To collect sufficient MSCs, 10% fetal bovine serum (FBS, HyClone, SH30070, Logan, UT, USA) was supplemented into Dulbecco’s modified Eagle’s medium (DMEM)/F12 medium (Gibco, 31,331,093, Grand Island, NY, USA) with 100 U/mL penicillin–streptomycin (PS, Gibco,10,378,016). Human Lung fibroblast cells (MRC5) were purchased from ATCC (ATCC, Manassas, VA, USA) and cultured in minimal Eagle’s Eagle medium (MEM, SH30024.01, HyClone) supplemented with 10% FBS and 100 U/mL PS. To collect MSCs-derived extracellular vesicles (MSCs-EVs) or MRC5-derived EVs from the relevant supernatant of MSCs or MRC5, cells were cultured in the medium supplemented with 10% EV-free FBS. MSCs or MRC5 involved in this study were from passage 3 to passage 8.

### Intravenous infusion of MSCs

MSCs were harvested and suspended in phosphate-buffered saline (PBS) at a final concentration of 5 × 10^5^/100 μL or 2.5 × 10^6^/100 μL stored in EP tube at 4 °C, respectively. Before intravenous infusion, MSCs were gently resuspended to keep them from aggregating. 200 μL PBS containing 1 × 10^6^ MSCs was injected into the mouse with a 28-gauge (28G) sterile needle via tail vein from Dox/Consecutive doses group as the pretreatment on days -3 and -1 followed by treatment on days 2, 5 and 8. Accordingly, 200 μL PBS containing 5 × 10^6^ MSCs was injected into the mouse from Dox/Single dose group on day 1 followed by 4 doses of 200 μL PBS on days -3, -1, 5, and 8. As for the Control or Dox group, 5 doses of 200 μL PBS were infused into mice at each time point.

### Construction of Dox-induced cardiotoxicity

Male CD1 mice (4–6 weeks) with an average bodyweight of 25–30 g were used in this study. All animals were purchased from Beijing Vital River Laboratories Company (Vital River, Beijing, China). Dox (cumulating dose 15 mg/kg of bodyweight, SFDA approval number H44024359, Shenzhen Main Luck Pharmaceuticals Inc, Shenzhen, China) was administrated intraperitoneally by 3 consecutive single injections of 5 mg/kg (0.5 mg/mL) every 2 days on days 0, 3, and 6. Mice were equally and randomly divided into Dox/PBS group, Dox/Single dose group and Dox/Consecutive doses group (*n* = 15 per group). Control mice (*n* = 15) received an equal volume of PBS intraperitoneally at each time point. Follow-up started from the first administration of Dox on day 0 and to 2 weeks. Bodyweight was recorded on days 0, 4, 7, 10, and 14. Mouse was euthanized as previously described, and the heart was isolated for further experiment. HW/BW was defined as the ratio of heart weight and bodyweight. The bodyweight at each time point minus bodyweight on day 0 was recorded as bodyweight loss. The percentage of bodyweight loss was defined as the ratio of bodyweight loss at each time point and bodyweight on day 0.

### Echocardiography

Standardized echocardiography technique was performed as previous literature described^.^ In brief, mouse was anesthetized according to previous description, and transthoracic echocardiography was performed on days 7 and 14 using an MS-250 ultrasound scanning transducer with VisualSonics Vevo 2100 machine (VisualSonics, Toronto, Canada). Two-dimensional images of left ventricular short-axis and long-axis projections were captured, and relevant M-mode tracing was recorded at the mid-papillary levels. Left ventricular ejection fraction (EF) and left ventricular fractional shortening (FS) were generated by VEVO computer algorithms.

### Measurement of serum myocardial biomarkers

Blood samples were collected from the angular vein of mice on days 7 and 14. After standing at room temperature (RT) for 30 min, blood samples were centrifuged at 3000 rpm for 15 min to isolate serum. Serum troponin T (TnT) or brain natriuretic peptide (BNP) was measured using ELISA assay kits (QIYI Biotech, QY-M30066, QY-M31187, Shanghai, China). All the procedures conformed to the manufacturer’s instructions.

### Histopathology

Heart or lung tissue was fixed in 4% paraformaldehyde (PFA) for 24 h and then routinely embedded in paraffin. For heart tissue, the paraffin section of ventricles was cut cross-sectionally into 5.0 μm, while 8-μm-thick coronal section was obtained from the embedded lung tissue. Both structure and morphology of ventricles or lungs were assessed with hematoxylin and eosin (H and E) staining. Moreover, collagen deposition in ventricles was detected by Sirius Red staining. Alexa Fluor 488 labeled wheat germ agglutinin (WGA, Invitrogen, W11261, Carlsbad, USA) was stained to identify the specific cardiac atrophy. All images were analyzed by FIJI ImageJ (https://imagej.net/software/fiji/). Quantitative analysis of the collagen deposition was performed as the percentage of positive staining area in the whole left ventricle area. The proportion of vacuolization in the ventricles was quantified as the ratio of the amount of vacuolization and cell nuclei.

### Isolation of cell and serum EVs

MSCs or MRC5 was cultured with respective medium supplemented with 10% EVs-removed FBS for 24–48 h. Before the next sub-cultivation, the cellular supernatant was collected and centrifuged at 500 g for 10 min to discard any cell contaminations. The supernatant was next centrifuged at 10,000 g for 30 min to remove cell debris and apoptotic bodies. Then, raw MSCs-EVs or MRC5-EVs was isolated by ultracentrifugation at 100,000 g for 70 min and washed by Dulbecco’s phosphate-buffered saline (DPBS). Finally, purified MSCs-EVs or MRC5-EVs was ultracentrifuged at 100,000 g for 2 h and the pellet was resuspended in PBS. All the procedures were performed at 4 °C.

Peripheral blood-derived EVs were isolated by Total Exosome Isolation (from serum) Kit (Invitrogen, 4,478,360) according to the manufacturer’s instructions. Briefly, 200 μL serum and 40 μL exosome isolation reagent was mixed by a pipette and vortexed until there is a homogenous solution, followed by the incubation for 30 min at 4 °C. Then, the pellet was centrifuged at 10,000 g for 10 min at RT and the supernatant was discarded. Finally, peripheral blood-derived EVs were collected and resuspended in a convenient volume of PBS. The total protein concentration of EVs was detected using a bicinchoninic acid (BCA) protein assay kit (Thermo Scientific, 23,225, Madison, WI, USA) following the manufacturer’s instructions.

### Transmission electron microscopy

To detect the morphological structure of MSCs-EVs and MRC5-EVs, a drop of EVs (1 μg/μL) was transferred onto a carbon film (Zhongjingkeji Technology, Beijing, China) with negative staining by 2% phosphotungstic acid. Next, transmission electron microscopy (TEM) images of MSCs-EVs and MRC5-EVs were captured from air-dried samples by the TEM (Talos F200C, MA, USA).

Preparation of mouse heart samples for TEM has been reported as previously described [31]. Briefly, excised hearts were cut into small tissue blocks (1 mm^3^) and fixed with 2.5% glutaraldehyde (Solarbio, P1126, Beijing, China) at 4 °C for 12–24 h and rinsed with PBS 3 times. Then, samples were postfixed in 2% osmium tetroxide (Sigma-Aldrich, 208,868, St Louis, MO, USA) for around 2 h, dehydrated in alcohol and infiltrated overnight with epoxy resin. Finally, after air dry for 48 h at 60 °C, samples were cut into an ultrathin section at 60 nm by an ultramicrotome (Reichert Ultracut, Vienna, Austria) followed by staining with uranyl acetate and lead citrate. The microstructure of ER in ventricular myocytes was captured by TEM.

### Particle size distribution of EVs

The particle size distribution of EVs was detected by dynamic light scattering (DLS) (Malvern Panalytical, Malvern, UK) as previously described [37].

### Neonatal mice cardiomyocytes culture

Primary mouse cardiomyocytes were isolated as previously described [38]. Ventricles of neonatal CD1 mice (< 24 h) were excised and rinsed by PBS plus 100 U/mL PS and then PBS, respectively. Ventricles were cut into small blocks and then enzymatically digested with 0.125% Trypsin (Gibco, 25,200,072) at 4 °C overnight. The cell suspension was collected after enzymatical digestion of collagenase II (Gibco, 17,101,015) at 37 °C for 2–4 min, and the single-cell suspension was obtained by passing a 70 μm cell filter and then cultured on the dish for 60 min. Nonattached cells were transferred and plated in Laminin (Thermo Scientific, 23,017,015)—coated dish supplemented with 1% ITS (Sigma-Aldrich, I3146) and 100 U/mL PS for 24 h. After that, neonatal cardiomyocytes were ready for further experiment.

### Doxorubicin treatment in vitro

Primary cardiomyocytes were seeded in 6-well plates and cultured in DMEM (Gibco, 11,965,092) supplemental with 1% ITS and 100 U/mL PS. Cardiomyocytes were pretreated with MSCs-EVs (100 μg/mL) for 6 h followed by adding 8 μM Dox for 24 h. As control, an equal volume of PBS replaces the MSCs-EVs and Dox. Morphology of cardiomyocytes was detected under the microscope (Olympus, Japan). To determine whether miR-181a-5p inhibited GRP78 expression in primary cardiomyocytes, 100 nmol/L miR-181a-5p mimics (RiboBio, Guangzhou, China) was transfected into cardiomyocytes by Lipofectamine 2000 (Lipo2000, Invitrogen, 11,668,500) for 24 h, and then, the cardiomyocytes were collected for the following experiment.

### Protein extraction and Western blotting

Ventricular tissue, cardiomyocytes, or EV pellets were lysed by radioimmunoprecipitation assay buffer (RIPA, Solarbio, R0010) for 30 min on ice along with vortex every 5 min. Total protein concentration of tissues, cells, or EVs was measured in line with the manufacturer’s instructions of BCA protein assay kit. Protein samples were separated by electrophoresis in SDS-PAGE gel with convenient concentrations based on the diverse molecular weight and then transferred into a polyvinylidene difluoride (PVDF) membrane (GE Healthcare, Milwaukee, MI, USA). The PVDF membrane was blocked with 5% skim milk followed by primary antibodies incubation overnight at 4 °C. The membrane was then incubated with the secondary antibodies after 3 times wash by TBST (5 min per time). The blots were exposed by adding ECL reagent (Millipore, WBULS0100, Burlington, MA, USA) and then imaged by gel doc XR system (Bio-Rad, Hercules, CA, USA). Antibodies involved in this study were listed as follows: Alix (WL03063, Wanleibio, China), CD63 (ab68418, Abcam, Cambridge, MA, USA), TSG101 (ab125011, Abcam), GRP78 (11,587-1-AP, Proteintech, Wuhan, China), IRE1α (#3294, Cell Signaling Technology, MA, USA), CHOP (15,204-1-AP, Proteintech), Caspase-12 (55,238-1-AP, Proteintech), Tubulin (ab6046, Abcam). The gray value of each blot was quantified by FIJI ImageJ.

### Reverse transcription and real-time quantitative polymerase chain reaction

Ventricular tissue, cells, or EVs were homogenized with TRIzol (Invitrogen, 15,596,018), and total RNA was isolated as described previously [16, 23]. Briefly, specific miRNA was reversely transcribed into first-strand cDNA using reverse transcriptase (Roche, 04,896,866,001, Mannheim, Germany) and Bulge-loop™ RT Primer (RiboBio). The first-strand cDNA and Bulge-loop™ Forward Primer (RiboBio), Bulge-loop™ Reverse Primers (RiboBio), Hieff™ qPCR SYBR Master Mix Kit (Yeasen, 11,202, Shanghai, China), and cDNA were mixed to perform quantitative real-time polymerase chain reaction (PCR). The relative expression of miRNAs was normalized by U6 (RiboBio). All the miRNA sequences are listed in Additional file 1: Table S2.

### Tracking transplanted MSCs fate in mice

To investigate transplanted MSCs retention in vivo, 5-day consecutive bioluminescence imaging (BLI) was performed in mice administered with a single dose of MSCs (5 × 10^6^ MSCs/dose) or consecutive doses of MSCs (1 × 10^6^ MSCs/dose). In summary, 2 consecutive doses of 1 × 10^6^ DF-MSCs for every 3 days were injected into mice through the tail vein on days 0 and 3, while a single dose of 5 × 10^6^ DF-MSCs was infused into mice on day 0. The distribution and signal intensity of *Fluc*-labeled MSCs were then measured every day using the IVIS 200 Series Imaging System (PerkinElmer, Waltham, MA, USA) with intraperitoneal administration of 25 μL D-Luciferin (1 μL/g) (Gold Biotechnology, eLUCK100, St. Louis, MO, USA). The signal intensity of *Fluc*-labeled MSCs was normalized by the signal value of mice treated with a single dose of wide-type MSCs (MSCs-WT). All analyses were performed using Living Image (Xenogen, Caliper LifeScience, Hopkinton, MA, USA).

To explore the colocalization of transplanted MSCs and the pulmonary vasculature, 3 consecutive doses of 1 × 10^6^ DF-MSCs were administered to mice by tail vein in 3 consecutive days. After final administration, tetramethylrhodamine isothiocyanate–Dextran (TRITC-Dextran, T1287, Sigma-Aldrich) was injected into mice via the tail vein to track pulmonary vasculature, and intravenous MSCs can be normally tracked by detection of enhanced eGFP. The lung was excised and placed on the stereomicroscope (Nikon, Japan) to capture relevant fluorescent images.

### Detection of serum EVs derived from MSCs

To track whether the intravenous infusion of MSCs secretes EVs into the blood, 3 consecutive doses (1 × 10^6^ MSCs/Dose) of Gluc-MSCs and MSCs-WT were intravenously injected into mice in 3 consecutive days. Blood was collected at 6 h after the final injection, and the total serum EVs were isolated from an equal volume of serum as previous described. The Gaussia luciferase activity in the total serum EVs was detected by Pierce™ Gaussia Luciferase Flash Assay Kit (16,158, Thermofisher) according to the manufacturer’s instructions.

### Isolation of miR-181a-5p knockdown and overexpressed MSCs-EVs

Knockdown or overexpression of miR-181a-5p in MSCs-EVs was performed as previous described [16, 39]. Briefly, 1 × 10^5^ MSCs were cultured into 6-well plates with DMEM medium (without PS and FBS). As confluence reached to around 70%, 200 nM miR-181a-5p Inhibitor (RiboBio), 200 nM miRNA Inhibitor negative control (NC) (RiboBio), 200 nmol/L miR-181a-5p Agomir (RiboBio), or 200 nM miRNA Agomir NC (RiboBio) were transfected into MSCs using Lipo2000, respectively. Cellular supernatant from each group was collected after being cultured for 72 h, and EVs were harvested in line with the above procedures.

### Immunofluorescence staining

For tissue samples, isolated heart or lung was fixed in 4% PFA for 6 h and embedded into OCT (Sakura Finetek, 4583, Japan), while additional cryoprotection of 30% sucrose solution was performed between tissue fix and OCT embedding. All samples were transversely cut into 8 μm sections. For cellular samples, primary cardiomyocytes were seeded on Laminin-coated slides followed by a fix of 4% PFA for 10 min on ice and 20 min at room temperature. Cellular samples were co-stained with anti-Rabbit GRP78 antibodies (11,587-1-AP, Proteintech) and anti-mouse Troponin I (TnI) antibodies (66,376-1-Ig, Proteintech) or anti-mouse alpha-actinin (Actinin, ab18061, Abcam). For Lung samples, anti-mouse PECAM-1 antibodies (550,274, BD Pharmingen™, NJ, USA) were stained to track pulmonary vasculature. Moreover, ventricular tissue was incubated with anti-Rabbit GRP78 antibody and anti-mouse TnI antibody. Nuclei of each sample were stained with 4′,6-diamidino-2-phenylindole (DAPI, C0065, Solarbio), and then, all samples on glass slides were mounted by Mounting Medium, antifading (Solarbio, S2100). Fluorescence images were captured by the fluorescent microscope (Olympus) or the confocal microscope (Olympus).

### TUNEL assay

To assess the apoptotic proportion of cardiomyocytes, terminal deoxynucleotidyl transferase dUTP nick-end labeling (TUNEL) assay was performed in line with the manufacturer’s instructions (Solarbio, T2190) followed by DAPI staining. The apoptotic proportion was quantified as the ratio of TUNEL-positive cells and total nuclei by FIJI ImageJ software.

### Flow cytometry

Single-cell suspension was collected from 6-well plates cultured with cardiomyocytes exposed on PBS, Dox/PBS, or Dox/MSCs-EVs, and FITC-conjugated Annexin V/propidium (PI, Solarbio, C1020) was stained to assess the apoptotic proportion of cardiomyocytes according to the manufacturer’s instructions. Apoptotic cells were identified in FITC^+^/PI^−^ regions as previous described [40]. Blood samples were collected after the final dose of 3 consecutive doses of intravenous DF-MSCs at 6 h, 12 h, and 24 h. All the serum EVs were collected from blood samples. Both cellular samples and EVs samples were detected by a FACS instrument. Data were analyzed by Flowjo™ X (BD Pharmingen™).

### Prediction of GRP78-targeted miRNAs

To explore potential GRP78-targeting miRNAs, 3 diverse miRNA datasheets, which predict miRNA-mRNA interactions, were searched online. Online websites were listed as follows: miRDB (http://www.mirdb.org) [41], TargetScan (http://www.targetscan.org) [42], and miRanda (http://www.miranda.org) [43]. All miRNAs were exported after inserting “mouse HSPA5” (GRP78 also was named as HSPA5) into these 3 databases. The potential miRNAs were only defined as overlapping in all 3 databases to target GRP78.

### Luciferase reporter assay

To confirm the miRNA-mRNA interaction between miR-181a-5p and GRP78, luciferase reporter assay was performed as previous described [29]. Briefly, 3’ UTR and antisense mutational 3’UTR fragments of GRP78 mRNA were inserted into PGL3-basic vectors (Promega, E1751, Madison, WI), which were termed as PGL3-basic GRP78-wt, and PGL3-basic GRP78-mut, respectively. MiR-181a-5p mimics (50 nmol/L, 100 nmol/L) or 100 nmol/L miRNA mimic NC was co-transfected jointly with pRL-TK vector (Promega, E2241) and 50 ng PGL3-basic GRP78-wt or PGL3-basic GRP78-mut, respectively, into HEK-293 T (293 T) cells by using Lipo2000. Meanwhile, miR-181a-5p Inhibitor (50 nmol/L, 100 nmol/L) or 100 nmol/L miRNA Inhibitor NC was co-transfected with pRL-TK vector and 50 ng PGL3-basic GRP78-wt or PGL3-basic GRP78-mut, respectively, into 293 T cells by Lipo2000. After 6 h incubation with transfection complex, fresh DMEM medium free with FBS and PS was replaced for the further 24 h incubation. Firefly or renilla luciferase activities were measured by the Dual-Lumi™ II Luciferase Reporter Assay kit (Beyotime, RG089S, Shanghai, China) in line with the manufacturer’s instructions. Luciferase activity of each sample was normalized by the ratio of renilla and firefly luciferase signals in 293 T cells.

### Statistical analysis

All data were expressed as Mean ± SD. Statistical analysis was performed with GraphPad prism 8.0 (GraphPad, CA, USA). Continuous data were presented as Mean ± SEM. The Kolmogorov–Smirnov test was performed to check the data distribution. The Levene test was performed to check the equality of variances. Unpaired two-tailed Student’s *t* tests were performed to analyze statistical significance between the two groups. One-way ANOVA tests were performed to compare the difference among multiple groups followed by Bonferroni post hoc tests for intraclass comparison. The survival curve was depicted by the Kaplan–Meier method followed by statistical analysis of the logarithmic rank test. The *P* value < 0.05 was considered as a significant statistical difference.

## Results

### Intravenously transplanted MSCs alleviated Dox-induced cardiotoxicity

To select an appropriate dose strategy to achieve the optimized therapeutic effect, we generated genetically modified MSCs with a double-fusion reporter gene (DF-MSCs) and injected a single dose (5 × 10^6^ cells, 1 dose) or consecutive dose of DF-MSCs (1 × 10^6^ cells, 5 doses) into mice intravenously. Using bioluminescence imaging (BLI), we found that DF-MSCs undergoing either cell delivery way were almost trapped and retained in the lung rapidly and steadily. Although the 1 × 10^6^ MSCs disappeared more quickly than the 5 × 10^6^ MSCs, the bioluminescence signal was much more intensified and prolonged after supplementing with the second dose of 1 × 10^6^ MSCs, suggesting that in the context of the same cell population, consecutive doses of MSCs prolonged the dwell time of viable MSCs in vivo (Additional file 1: Fig. S1). This prolonged cell retention of intravenously infused MSCs may be the biological basis of the enhanced therapeutic ability.

**Fig. 1:**
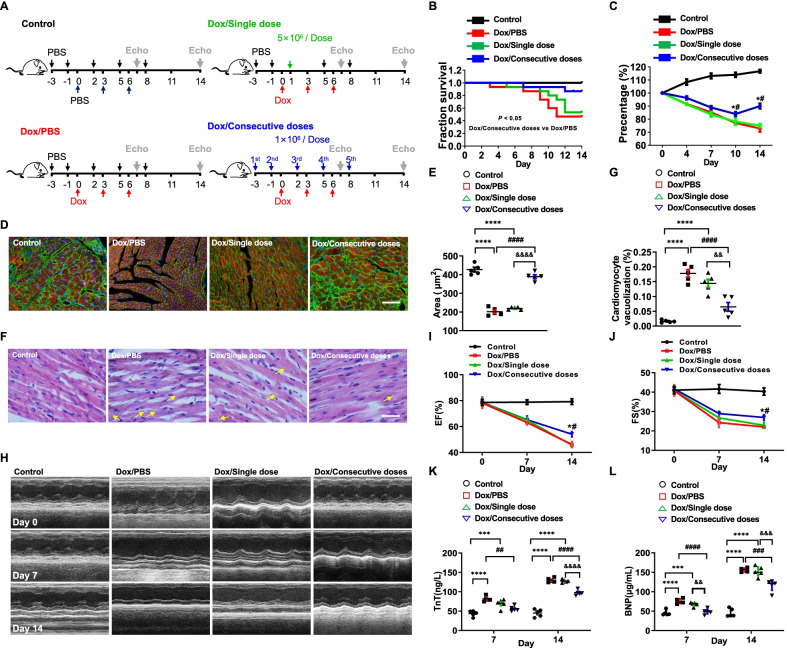
Consecutive MSC infusion alleviated Dox-induced cardiotoxicity. **A.** Experimental scheme of this study design. **B.** Survival analysis of mice administrated with PBS (Control), Dox/PBS, Dox/Single dose, or Dox/Consecutive doses (*n* = 15). *P* < 0.05, Dox/Consecutive doses vs Dox/PBS. Data were analyzed by two-sided Kaplan–Meier estimator. **C.** Quantitative proportion of bodyweight loss in mice administrated with PBS (Control), Dox/PBS, Dox/Single dose, or Dox/Consecutive doses, on days 0, 4, 7, 10, and 14 (*n* = 4). ^*^P < 0.05 vs Dox/PBS, ^#^*P* < 0.05 vs Dox/Single dose. **D** and **E.** Representative immunofluorescence images of ventricles and statistics of cell cross-sectional area (*n* = 5) in mice administrated with PBS (Control), Dox/PBS, Dox/Single dose, or Dox/Consecutive doses. Scale bar, 100 μm. ^****^P < 0.0001 vs Control, ^####^*P* < 0.0001 vs Dox/PBS, ^&&&&^*P* < 0.0001 vs Dox/Single dose. **F** and **G.** Representative ventricular structure and quantitative vacuolization (*n* = 5) in mice administrated with PBS (Control), Dox/PBS, Dox/Single dose or Dox/Consecutive doses. Scale bar, 50 μm. ^****^P < 0.0001 vs Control, ^####^*P* < 0.0001 vs Dox/PBS, ^&&^*P* < 0.01 vs Dox/Single dose. **H.** Representative echocardiographic images (M-mode) of mice administered with PBS (Control), Dox/PBS, Dox/Single dose or Dox/Consecutive doses on days 0, 7 and 14. **I** and **J.** Statistics of the left ventricular ejection fraction (EF) and fractional shortening (FS) in each group on days 0, 7 and 14 (*n* = 5). ^*^P < 0.05 vs Dox/PBS, ^#^*P* < 0.05 vs Dox/Single dose. **K** and **L.** Serum levels of brain natriuretic peptide (BNP) and troponin T (TnT) in mice administrated with PBS (Control), Dox/PBS, Dox/Single dose or Dox/Consecutive doses on days 7 and 14 (*n* = 5). ^***^P < 0.001, ^****^P < 0.0001 vs Control, ^##^*P* < 0.01, ^###^*P* < 0.001, ^####^*P* < 0.0001 vs Dox/PBS, ^&&&^*P* < 0.001, ^&&&&^*P* < 0.0001 vs Dox/Single doses. Data were analyzed by one-way ANOVA followed by Bonferroni post hoc test, unless specifically indicated

We next designed this research scheme (Fig. 1A). A single dose of MSCs (5 × 10^6^ cells per dose) was infused into the mice through the tail vein, while in the Dox/Consecutive doses group, 5 consecutive doses of MSCs (1 × 10^6^ cells per dose) were injected at different 5 time points. Dox shortened the survival period of the mice and contributed to continuous bodyweight loss, while only consecutive infusion of MSCs prolonged the survival period and terminated the bodyweight loss of the mice (Fig. 1B, C). Due to being trapped in the lung, further observation indicated that no extra lung injury was found in mice administered with either cell infusion (Additional file 1: Fig. S2). Collectively, consecutive doses of MSCs improve the general condition of mice exposed to Dox but do not injure the lung.

We then performed pathological assessments, echocardiography, and cardiac biomarker tests. The cross-sectional area of the myocytes was measured to quantify the extent of cardiac atrophy (Fig. 1D, E). The myocyte size was reduced after being injected with Dox on day 14 but enlarged with consecutive doses of MSCs. In addition, consecutive doses of MSCs also decreased the proportion of myocyte vacuolization in mice administrated with Dox, while there was no significant difference between Dox/PBS group and PBS/Single dose group (Fig. 1F, G). The ventricular structure and the ratio of heart weight and bodyweight (HW/BW) were also detected and indicated Dox hardly changed HW/BW on day 7 but significantly decreased the HW/BW value on day 14. Either dose strategy increased HW/BW, while of them, consecutive doses of MSCs exhibited a better effect than a single dose of MSCs (Additional file 1: Fig. S3A). Dilated left ventricle and thinner ventricular muscles were observed in mice exposed to Dox on day 14, while more minor changes in mice jointly administered with consecutive doses of MSCs (Additional file 1: Fig. S3B). Taken together, consecutive doses of MSCs attenuate cardiac morphological damage better.

Left ventricular dysfunction and myocardial injury are critical clinical characteristics in Dox-induced cardiotoxicity. We found the left ventricular ejection fraction (EF) and fractional shortening (FS) decreased since day 7, only consecutive doses of MSCs improved these parameters compared to a single dose of MSCs or PBS on day 14 (Fig. 1H–J). Then, serum TnT and BNP were increased in mice administrated with Dox since day 7 with comparable levels in mice jointly administrated with a single dose of MSCs or PBS. Dramatical decreases in serum TnT and BNP were observed in mice jointly administrated with consecutive doses of MSCs (Fig. 1K, L). Finally, Sirius Red staining showed only slight fibrotic deposition in mice administered with Dox, but reduced fibrotic accumulation was still observed in mice jointly administered with consecutive doses of MSCs (Additional file 1: Fig. S4). Taken together, consecutive doses of MSCs improve left ventricular systolic function and reduce abnormal fibrotic accumulation in Dox-induced cardiotoxicity.

### Intravenously transplanted MSCs attenuated ER stress-induced apoptosis

**Fig. 2:**
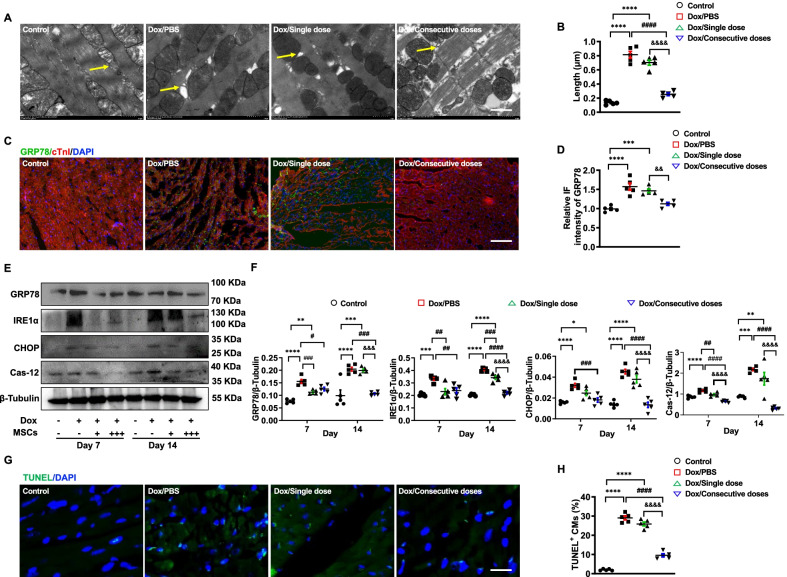
Intravenously transplanted MSCs attenuated endoplasmic reticulum stress-induced apoptosis. **A** and **B.** Representative TEM images and long-axis length of dilated endoplasmic reticulum (ER) of ventricular tissue in mice administered with PBS (control), Dox/PBS, Dox/Single dose, or Dox/Consecutive doses on day 14. The dilated ER was identified by a yellow arrow. Scale bar, 1 μm, (*n* = 5). ^****^P < 0.0001 vs Control, ^####^*P* < 0.0001 vs Dox/PBS, ^&&&&^*P* < 0.0001 vs Dox/Single dose. **C.** Representative immunofluorescence images of cardiac troponin I (cTnI, red) and GRP78 antibody (green) binding to ventricular tissue of mice administered with PBS (control), Dox/PBS, Dox/Single dose, or Dox/Consecutive doses on day 14. Scale bar, 100 μm. **D.** Statistics of the fluorescence intensity of GRP78 in each group on day 14 (*n* = 5). ^***^P < 0.001, ^****^P < 0.0001 vs Control, ^&&^*P* < 0.01 vs Dox/Single dose. **E** and **F.** Western Blotting and quantified protein expression of the ER stress-induced apoptosis pathway in ventricular tissue of mice administrated with PBS (Control), Dox/PBS, Dox/Single dose on days 7 and 14, (*n* = 5). ^*^P < 0.05, ^**^P < 0.01, ^***^P < 0.001, ^****^P < 0.0001 vs Control, ^#^*P* < 0.05, ^##^*P* < 0.01, ^###^*P* < 0.001, ^####^*P* < 0.0001 vs Dox/PBS, ^&&&^*P* < 0.001, ^&&&&^*P* < 0.0001 vs Dox/Single dose. **G** and **H.** TUNEL assay (green) and apoptotic proportion of ventricular tissue in mice administrated with PBS (Control), Dox/PBS, Dox/Single dose, or Dox/Consecutive doses on day 14. Scale bar, 50 μm (*n* = 5). ^****^P < 0.0001 vs Control, ^####^*P* < 0.0001 vs Dox/PBS, ^&&&&^*P* < 0.0001 vs Dox/Single dose. Data were analyzed by one-way ANOVA followed by Bonferroni post hoc test, unless specifically indicated

ER morphology of cardiomyocytes imaged by transmission electron microscopy (TEM) showed a large amount of dilated ER in mice exposed to Dox. However, consecutive doses of MSCs decreased the proportion of dilated ER significantly, whereas such a positive effect could not be detected in mice jointly administered with a single dose of MSCs (Fig. 2A, B). To evaluate whether such dilated ER contributes to cell apoptosis triggered by upregulated GRP78, we next measured the expression of GRP78 in ventricular tissue. Our result showed that significant upregulation of GRP78 could be observed in the ventricle once administered with Dox regardless of whether a single dose of MSCs was administered. Interestingly, such GRP78 expression was dramatically decreased in mice jointly administered with consecutive doses of MSCs, when compared with PBS or a single dose of MSCs (Fig. 2C, D).

Then, we detected the expression of key pathway proteins in ER stress-induced apoptosis, which showed entire key protein levels were upregulated in this signaling pathway from day 7 in Dox-administrated mice. However, consecutive doses of MSCs are more capable of inhibiting the upregulation on day 14 (Fig. 2E, F). Finally, we examined the apoptotic proportion of ventricular cells in each group on day 14, which is parallel to the Western Blotting data. Administration of Dox increased the proportion of cell apoptosis and only jointly administering consecutive doses of MSCs attenuated the cell apoptosis in the myocardium (Fig. 2G, H). Taken together, our observations suggested the cardioprotective effect of intravenously transplanted MSCs on Dox-induced cardiotoxicity could result from the suppression of ER stress-induced apoptosis.

### MSC-EVs downregulated ER stress-induced apoptosis in cardiomyocytes

**Fig. 3:**
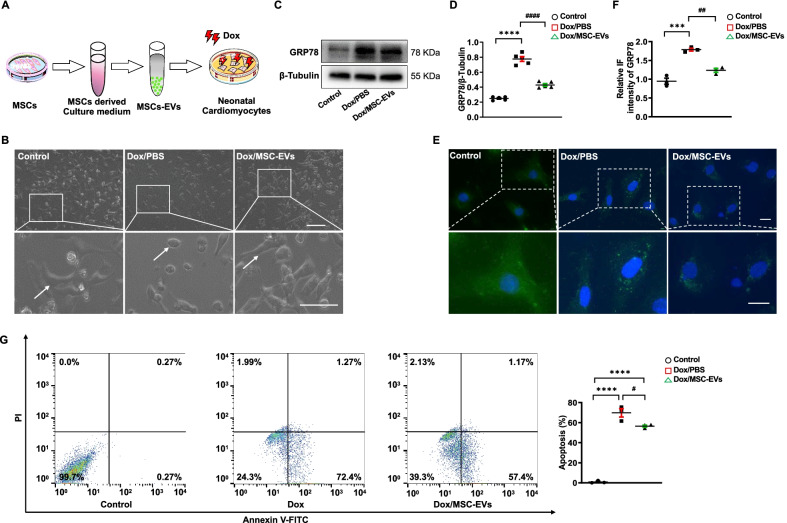
MSC-derived extracellular vesicles decline ER stress-induced apoptosis in cardiomyocytes. **A.** Experimental scheme of the design of the in vitro study. Extracellular vesicles derived from MSCs (MSC-EVs, 200 μmol/L), Dox (8 μmol/L). **B.** Representative bright-field images of cardiomyocytes exposed to PBS (control), Dox/PBS, or Dox/MSC-EVs. Injured cardiomyocytes were identified by bright arrows. Scale bar, 100 μm. **C** and **D.** Western Blotting and quantified GRP78 expression in cardiomyocytes exposed to PBS (Control), Dox/PBS, or Dox/MSC-EVs (*n* = 5). ^****^P < 0.0001 vs Control, ^####^*P* < 0.0001 vs Dox/PBS. **E.** Representative immunofluorescence images of antibody to GRP78 (green) binding to cardiomyocytes exposed to PBS (Control), Dox/PBS, or Dox/MSC-EVs. Scale bar, 25 μm. **F.** Statistics of the fluorescence intensity of GRP78 in each group (*n* = 3). ^***^P < 0.001 vs Control, ^##^*P* < 0.01 vs Dox/PBS. **G.** Representative Annexin V-FITC/PI staining and quantitative apoptotic proportion of cardiomyocytes exposed to PBS (control), Dox/PBS, or Dox/MSC-EVs (*n* = 3). ^****^P < 0.0001 vs Control, ^#^*P* < 0.05 vs Dox/PBS. Data were analyzed by one-way ANOVA followed by Bonferroni post hoc test, unless specifically indicated

In myocardium, ER stress-induced apoptosis was increased after administrated with Dox, and we next investigated whether this kind of cell apoptosis could be observed in the cardiomyocytes or not (Fig. 3A). The undetectable retention of MSCs in the heart urges us to hypothesize that MSC-EVs may be the link between the lung-trapped MSCs and the injured myocardium. Therefore, we isolated MSC-EVs and added them to primary mouse cardiomyocytes exposed to Dox. Initially, MSC-EVs were collected and characterized by a typical sphere-shaped bilayer membrane structure, approximately size distribution of EVs, and certain protein expression (Additional file 1: Fig. S5). Then, after being exposed to Dox and/or MSC-EVs, the morphology of cardiomyocytes was observed by bright-field images, which revealed that cardiomyocytes lost their normal morphology as exposed to Dox, while MSC-EVs were conducive to maintaining this normal morphology (Fig. 3B).

Next, the expression of GRP78 and the apoptotic proportion of cardiomyocytes were detected to highlight that ER stress was overactivated followed by cardiomyocyte apoptosis. The results showed that GRP78 expression was increased in cardiomyocytes exposed to Dox but decreased as jointly treated with MSC-EVs (Fig. 3C, D). Furthermore, immunofluorescence staining confirmed that this upregulated GRP78 was weakened by supplementing with MSC-EVs (Fig. 3E, F). Further detection of apoptosis showed increased cardiomyocyte apoptosis as exposed to Dox but significantly declined as supplementing with MSC-EVs (Fig. 3G). Taken together, our findings indicated that Dox activated ER stress and induced cardiomyocyte apoptosis, and MSC-EVs had a protective effect in suppressing this ER stress-induced cardiomyocyte apoptosis.

### MiR-181a-5p-enriched MSC-EVs targeted and inhibited GRP78

**Fig. 4:**
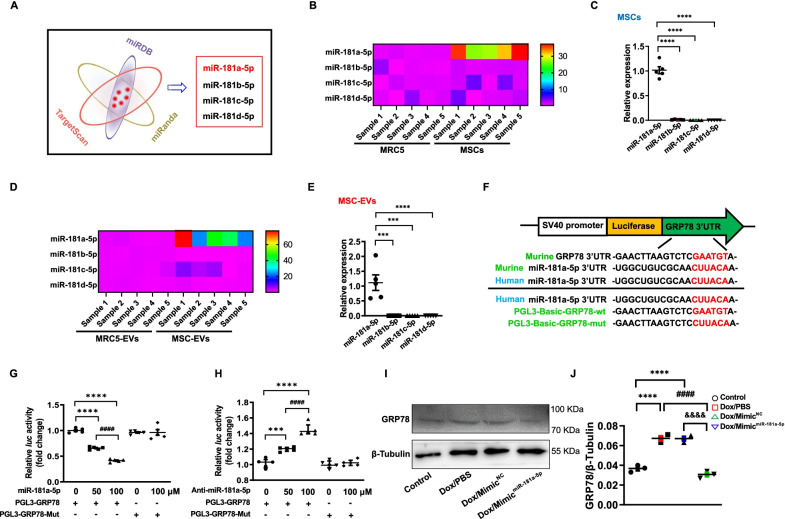
MiR-181a-5p-enriched MSC-EVs inhibit the expression of GRP78. **A.** Schematic illustration of filtrating potential GRP78-targeting miRNAs from TargetScan, miRanda, and miRDB. **B.** Heat map of miRNAs profiles in MSCs and MRC5. **C.** Relative miRNA levels in MSCs (*n* = 5). ^****^P < 0.0001 vs miR-181b-5p, miR-181c-5p, and miR 181d-5p. **D.** Heat map of miRNA profiles in MSC-EVs and MRC5-EVs. **E.** Relative expression of miRNAs in MSC-EVs (*n* = 5). ^***^P < 0.001, ^****^P < 0.0001 vs miR-181b-5p, miR-181c-5p, and miR 181d-5p. **F.** Schematic design of the GRP78 labeled double-luciferase reporter system. **G.** The inhibiting efficacy of miR-181a-5p on GRP78 (*n* = 5). ^****^P < 0.0001 vs miR-181a-5p (0 μmol/L). ^####^*P* < 0.0001 vs miR-181a-5p (50 μmol/L). **H.** The intensifying efficacy of anti-miR-181a-5p in GRP78 (*n* = 5). ^***^P < 0.001, ^****^P < 0.0001 vs anti-miR-181a-5p (0 μmol/L). ^####^*P* < 0.0001 vs anti-miR-181a-5p (50 μmol/L). **I** and **J.** Western Blotting and quantitative GRP78 expression in cardiomyocytes exposed to PBS (Control), Dox/PBS, Dox/Mimic^NC^, and Dox/Mimic^miR−181a−5p^, (*n* = 3). ^****^P < 0.0001 vs Control, ^####^*P* < 0.0001 vs Dox/PBS. ^&&&&^*P* < 0.0001 vs Dox/Mimic.^NC^. Data were analyzed by one-way ANOVA followed by Bonferroni post hoc test, unless specifically indicated

Dox-induced cardiotoxicity was a consequence of ER stress-induced cardiomyocyte apoptosis, which was triggered by upregulated GRP78. Therefore, our research interests then concentrated on which molecules from MSC-EVs downregulated GRP78 in the ventricle. A recent study reported that EVs derived from multiple cell types encapsulating a wide range of miRNAs contribute to intercellular communication by targeting and inhibiting post-transcription processing of certain proteins [11]. To determine the specific bioactive components encapsulated in MSC-EVs, which could target and inhibit GRP78, we used bioinformatic analysis to predict potential miRNAs targeting GRP78 from online miRNA datasheets (Additional file 1: Table S1). Our data showed that only miR-181a-5p, miR-181b-5p, miR-181c-5p, and miR-181d-5p were included in the three datasheets simultaneously (Fig. 4A, Additional file 1: Table S1). As the majority of intravenously injected MSCs were trapped in the lung, we selected a lung tissue-derived cell type (MRC5) as a negative control to screen out the potential GRP78-targeting miRNA. MRC5-EVs were collected from the supernatant of MRC5 and identified with typical characteristics of EVs (Additional file 1: Fig. S5). We found that MSCs expressed miR-181a-5p about 30 times higher than MRC5, and there was also around 40 times higher expression in MSC-EVs compared to MRC5-derived EVs (MRC5-EVs). No statistical or reverse differences in miRNAs were found between MSCs and MRC5 or between MSC-EVs and MRC5-EVs (Fig. 4B, D, Additional file 1: Fig. S6). Next, we measured the abundance of these four miRNAs in MSCs or MSC-EVs. As expected, miR181a-5p expressed the highest level in MSCs and MSC-EV compared to the other candidates (Fig. 4C, E). The detailed miRNA sequences of human and mouse are exhibited in Additional file 1: Table S2.

To validate the targeting and inhibiting efficacy of miR181a-5p on GRP78, we constructed a double-luciferase reporter system. 3’UTR of GRP78 mRNA or the antisense mutational 3’UTR of GRP78 mRNA was cloned into the PGL3-basic vector, which was termed PGL3-basic GRP78-wt or PGL3-basic GRP78-mut (Fig. 4F). Our luciferase reporter gene assay elucidated that miR-181a-5p significantly reduced luciferase activity by targeting the 3’UTR of GRP78 mRNA in HEK-293 T cells as transfecting with the PGL3-basic GRP78-wt vector but did not decrease luciferase activities of 293 T cells with the PGL3-basic GRP78-mut vector (Fig. 4G). Furthermore, by adding anti-miR-181a-5p, the luciferase activities of 293 T cells transfected with the GRP78-wt PGL3-basic vector were significantly increased, but there was no difference in 293 T cells transfected with the GRP78-mut vector PGL3-basic (Fig. 4H). Finally, we detected GRP78 expression in cardiomyocytes exposed to Dox and miR-181a-5p Mimic, which found a decrease in GRP78 expression at protein level (Fig. 4I, J). Taken together, current evidence supported that MSCs released miR-181a-5p-enriched MSC-EVs, which could efficiently target and inhibit GRP78.

### MiR-181a-5p-enriched MSC-EVs were transferred to the myocardium through blood

**Fig. 5:**
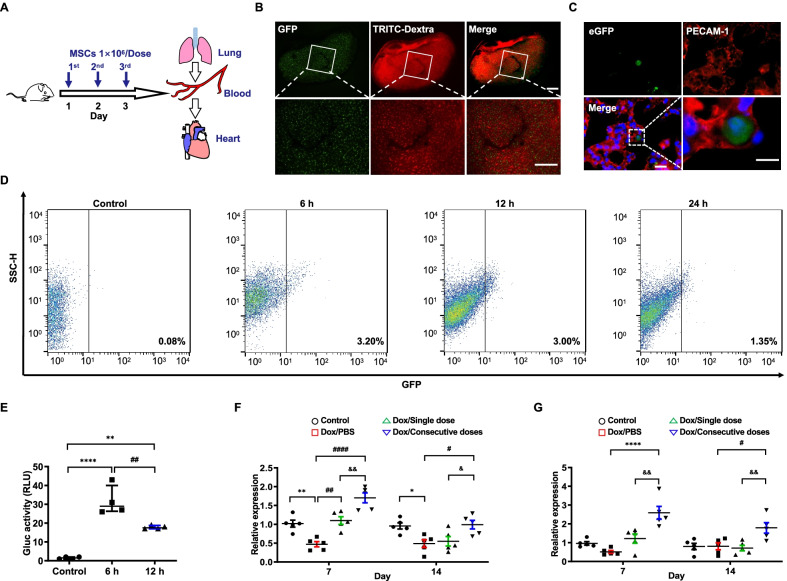
Lung-trapped MSCs secrete miR-181a-5p-enriched MSC-EVs into the myocardium through blood. **A.** Experimental scheme of MSC tracking in vivo. **B.** Representative stereomicroscopic images of lung tissue in mice administered with MSCs intravenously. Scale bar, 2 mm. **C.** Representative immunofluorescence images of eGFP (green) and PECAM-1 antibody (red) binding to lung tissue in mice intravenously administered with MSCs on 24 h. Scale bars, 50 μm. **D.** Quantitative proportion of eGFP^+^ EVs in serum EVs from mice injected with MSCs-WT or DF-MSCs (6 h, 12 h and, 24 h). **E.** Gluc activity of serum EVs from mice injected with MSCs-WT and Gluc-MSCs (6 h and 12 h) (*n* = 3) ^**^P < 0.01, ^****^P < 0.0001 vs PBS, ^##^*P* < 0.01 vs 6 h. **F.** MiR-181a-5p levels of serum EV in mice administrated with PBS (control), Dox/PBS, Dox/Single dose, or Dox/Consecutive doses on days 7 and 14 (*n* = 5). ^*^P < 0.05, ^**^P < 0.01 vs PBS (Control), ^#^*P* < 0.05, ^##^*P* < 0.01, ^####^*P* < 0.0001 vs Dox/PBS, ^&^*P* < 0.05, ^&&^*P* < 0.01 vs Dox/Single dose. **G.** MiR-181a-5p levels of ventricular tissue in mice administrated with PBS (Control), Dox/PBS, Dox/Single dose, or Dox/Consecutive doses on days 7 and 14 (*n* = 5). ^****^P < 0.0001 vs PBS (Control), ^#^*P* < 0.05 vs Dox/PBS, ^&&^*P* < 0.01, ^&&&&^*P* < 0.0001 vs Dox/Single dose. Data were analyzed by one-way ANOVA followed by Bonferroni post hoc test, unless specifically indicated

To describe how intravenously infused MSCs affected the injured myocardium from lung to heart, we designed this experimental scheme (Fig. 5A). Three consecutive doses of wild-type MSCs (WT-MSCs), DF-MSCs, and Gluc-MSCs were injected intravenously in 3 days, and the main organs and peripheral blood were collected, respectively, and analyzed later. Our BLI results showed that intravenously injected DF-MSCs were almost trapped in the lung **(**Additional file 1: Fig. S1). As Dextran binding to TRITC was injected into mice after the final dose, transplanted DF-MSCS centered on the TRITC binding area were trapped in the terminal pulmonary capillary network (Fig. 5B). To observe the precise location between DF-MSCs and the capillary lumen, immunofluorescence staining of PECAM-1, which is a typical marker of vascular endothelial cells, was performed to confirm the spatial relationship with the enhanced green fluorescence protein (eGFP^+^) and DF-MSCs. The results showed that intravenously infused DF-MSCs were gathered in the capillary lumen for at least 24 h (Fig. 5C). This consistent retention in the pulmonary capillary lumen facilitated the release of EVs into the blood. To validate that intravenously injected MSCs secrete EVs in blood, two types of genetically modified MSCs were administered to mice including DF-MSCs and Gluc-MSCs compared to MSCs-WT as a negative control (Fig. 5A). For Gluc-MSCs, significant Gluc activity could be detected in EVs derived from Gluc-MSCs as previously described, and for DF-MSCs, we detected positive eGFP signals in EVs derived from DF-MSCs by FACS (Additional file 1: Fig. S7). We collected serum from mice administered with WT-MSCs, DF-MSCs, or Gluc-MSCs. EGFP^+^ EVs were detected in serum from mice administered with DF-MSCs compared to MSCs-WT. The proportion of eGFP^+^ EV reached the maximum level at 6 h and then gradually decreased over time. Next, we tested Gluc activity in serum collected from mice administered with Gluc-MSCs and WT-MSCs, which showed increased signals in serum from mice administered with Gluc-MSCs (Fig. 5E). Collectively, these results suggest that intravenously infused MSCs could release detectable EVs into the blood.

As a slow and continuous delivery from the blood to the myocardium, MSC-EVs are difficult to be tested in the myocardium, and we concentrated on the therapeutic component inner MSC-EVs by quantitative PCR. We initially detected relative expression of miR-181a-5p of total serum EVs, myocardium, and vital organs in mice administered with MSCs or PBS, and the results showed significant increases in miR-181a-5p in the myocardium, serum EVs, and lung, but no difference in liver, kidney, and spleen (Additional file 1: Fig. S8). Furthermore, in the murine model, miR-181a-5p expression of serum EVs and myocardium was increased in mice jointly administrated with Dox and consecutive doses of MSCs from day 7 to day 14 (Fig. 5F, G). Taken together, these results suggest that MSC-EVs are released into the blood, increasing miR-181a-5p expression in serum EVs and myocardium.

### MiR-181a-5p inhibitor deteriorated Dox-induced cardiomyocyte damage

**Fig. 6:**
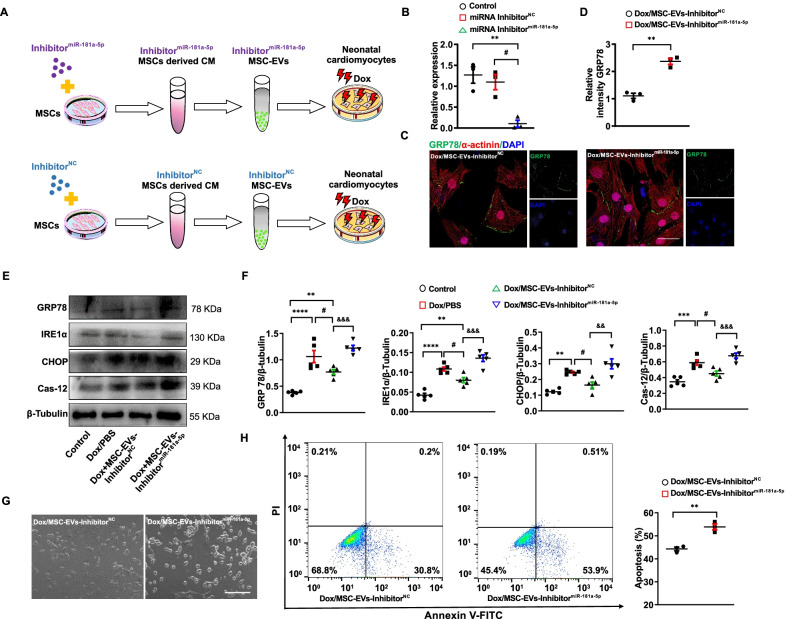
The MiR-181a-5p inhibitor weakens the cardioprotective ability of MSCs. **A.** Experimental design of in vitro study with the miR181a-5p inhibitor. **B.** Statistics of miR-181a-5p expression in cardiomyocytes exposed to PBS (Control), Inhibitor^NC^, or Inhibitor^miR−181a−5p^ (*n* = 3). ^**^P < 0.01 vs Control. ^#^*P* < 0.05 vs Inhibitor^NC^. Data were analyzed by one-way ANOVA followed by Bonferroni post hoc test, unless specifically indicated. **C.** Representative immunofluorescence images of antibody to α-actinin (Red) and GRP78 (green) binding to cardiomyocytes exposed to Dox/MSC-EVs-Inhibitor^NC^, or Dox/MSC-EVs-Inhibitor^miR−181a−5p^. Scale bar, 50 μm. **D.** Statistics of GRP78 expression in cardiomyocytes exposed to Dox/MSC-EVs-Inhibitor^NC^, or Dox/MSC-EVs-Inhibitor^miR−181a−5p^ (*n* = 3). ^**^P < 0.01 vs Dox/MSC-EVs-Inhibitor^NC^. Data were analyzed by unpaired two-tailed Student’s *t *test. **E.** Western Blotting and quantitative protein expression showing stress-induced apoptosis in cardiomyocytes exposed to PBS (Control), Dox/PBS, Dox/EVs-Inhibitor^NC^, or Dox/EVs-Inhibitor^miR−181a−5p^, (*n* = 5). ^**^P < 0.01, ^***^P < 0.001, ^****^P < 0.0001 vs Control, ^#^*P* < 0.05 vs Dox/PBS, ^&&^*P* < 0.01, ^&&&^*P* < 0.001 vs Dox/MSC-EVs-Inhibitor^NC^. Data were analyzed by one-way ANOVA followed by Bonferroni post hoc test, unless specifically indicated. **G.** Representative bright-field images of cardiomyocytes exposed to Dox/MSC-EVs-Inhibitor^NC^, or Dox/MSC-EVs-Inhibitor^miR−181a−5p^. Scale bar, 100 μm. **H.** Representative Annexin V-FITC/PI FACS staining and quantitative apoptotic proportion of cardiomyocytes exposed to Dox/MSC-EVs-Inhibitor^NC^, or Dox/MSC-EVs-Inhibitor^miR−181a−5p^ (*n* = 3). ^**^P < 0.01 vs Dox/MSC-EVs-Inhibitor^NC^. Data were analyzed by unpaired two-tailed Student’s *t *test

Current evidence indicated that intravenously injected MSCs secreted miR-181a-5p-enriched EVs into the myocardium through peripheral blood. MiR181a-5p-enriched MSC-EVs alleviated ER stress-induced apoptosis by suppressing GRP78. To provide further insight into this critical role of miR-181a-5p-enriched EVs in Dox-induced cardiomyopathy, we generated miR-181a-5p knockdown MSC-EVs (MSC-EVs-Inhibitor^miR−181a−5p^) or MSC-EVs-Inhibitor^NC^ by transfecting miR-181a-5p Inhibitor or miRNA inhibitor^NC^ into MSCs (Fig. 6A). MiR-181a-5p was significantly decreased in MSC-derived EVs collecting from supernatant cultured with MSCs supplementing with the miR-181a-5p inhibitor but not the miRNA inhibitor^NC^, indicating that the miR-181a-5p inhibitor had a considerable effect on downregulating miR-181a-5p within MSC-EVs (Fig. 6B). Next, we found that GRP78 expression was higher after adding MSC-EVs-Inhibitor^miR−181a−5p^ into cardiomyocytes (Fig. 6C, D). Then, we investigated whether this higher expression of GRP78 further affected the ER stress-induced apoptotic pathway. We showed that MSC-EVs-Inhibitor^NC^ suppressed the ER stress-induced apoptotic pathway in cardiomyocytes exposed to Dox, while MSC-EVs-Inhibitor^miR−181a−5p^ could further re-activate the whole pathway (Fig. 6E, F). Furthermore, the loss of normal morphology was much more severe in cardiomyocyte joints exposed to Dox and MSC-EVs-Inhibitor^miR−181a−5p^ (Fig. 6G). Finally, to verify this effect on the apoptosis promoted by ER stress, we sought to calculate the apoptotic proportion of cardiomyocytes exposed to Dox and MSC-EVs-Inhibitor^miR−181a−5p^ or MSC-EVs-Inhibitor^NC^. Cardiomyocytes exposed to Dox and MSC-EVs-Inhibitor^miR−181a−5p^ had a much higher apoptotic proportion (Fig. 6H). Taken together, our findings demonstrate that the cardioprotective effects of MSC-EVs on Dox-induced cardiomyocyte damage could be weakened by suppressing miR-181a-5p expression in MSC-EVs by miR-181a-5p inhibitor.

### MiR-181a-5p Agomir attenuated Dox-induced cardiomyocyte damage

**Fig. 7:**
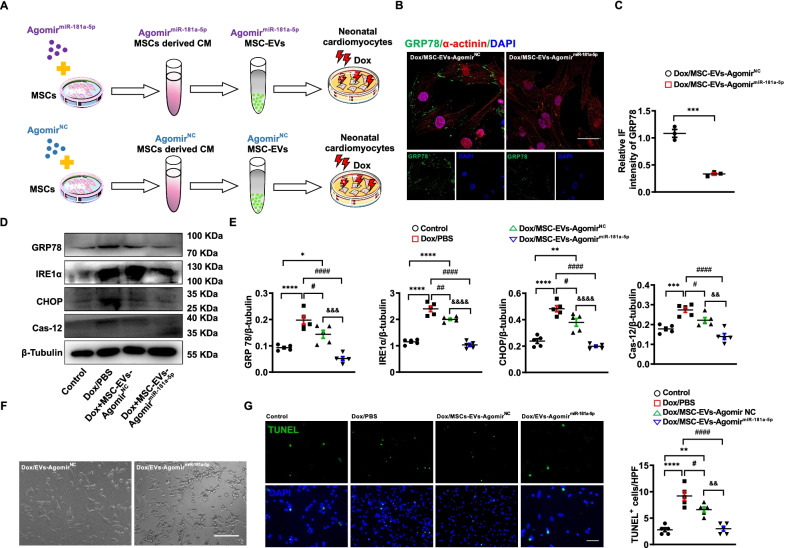
MiR-181a-5p Agomir improves the cardioprotective ability of MSCs. **A.** Experimental design of an in vitro study with miR181a-5p Agomir. **B.** Representative immunofluorescence images of antibody to α-Actinin (Red) and GRP78 (green) binding to cardiomyocytes exposed to Dox/MSC-EVs-Agomir^NC^, or Dox/ MSC-EVs-Agomir^miR−181a−5p^. Scale bar, 50 μm. **C.** Statistics of GRP78 expression of cardiomyocytes exposed to Dox/MSC-EVs-Agomir^NC^, or Dox/MSC-EVs-Agomir^miR−181a−5p^ (*n* = 3). ^***^P < 0.001 vs Dox/MSC-EVs-Agomir^NC^. Data were analyzed by unpaired two-tailed Student’s *t-*test. **D** and **E.** Western blotting and quantitative protein expression of ER stress-induced apoptosis in cardiomyocytes exposed to PBS (Control), Dox/PBS, Dox/MSC-EVs-Agomir^NC^, or Dox/MSC-EVs-Agomir^miR−181a−5p^, (*n* = 5). ^*^P < 0.05, ^**^P < 0.01, ^***^P < 0.001, ^****^P < 0.0001 vs Control, ^#^*P* < 0.05, ^##^*P* < 0.01, ^####^*P* < 0.0001 vs Dox/PBS, ^&&^*P* < 0.01, ^&&&^*P* < 0.001, ^&&&&^*P* < 0.0001 vs Dox/MSC-EVs-Agomir^NC^. Data were analyzed by the one-way analysis of variance followed by Bonferroni post hoc test. **F.** Representative bright-field images of cardiomyocytes exposed to Dox/MSC-EVs-Agomir^NC^, or Dox/MSC-EVs-Agomir^miR−181a−5p^. Scale bar, 100 μm. **G.** Representative images and quantitative proportion of TUNEL-positive (green) cardiomyocytes exposed to PBS (Control), Dox/PBS, Dox/MSC-EVs-Agomir^NC^, or Dox/MSC-EVs-Agomir^miR−181a−5p^. Scale bar, 50 μm (*n* = 5). ^**^P < 0.01, ^****^P < 0.0001 vs Control, ^#^*P* < 0.05, ^####^*P* < 0.0001 vs Dox/PBS, ^&&^*P* < 0.01 vs Dox/MSC-EVs-Agomir^NC^. Data were analyzed by one-way ANOVA followed by Bonferroni post hoc test, unless specifically indicated

The suppression of miR-181a-5p in MSC-EVs weakened the cardioprotective ability of MSC-EVs. Based on that, we further investigated whether upregulation of miR-181a-5p expression in MSC-EVs by miR-181a-5p Agomir (MSC-EVs-Agomir^miR−181a−5p^) could intensify cardioprotective effects on Dox-induced cardiotoxicity (Fig. 7A). MiR-181a-5p Agomir or miRNA Agomir^NC^ was transfected into MSCs to collect MSC-EVs-Agomir^miR−181a−5p^ or MSC-EVs Agomir^NC^. Our results revealed that GRP78 expression was further reduced after adding MSC-EVs-Agomir^miR−181a−5p^ into cardiomyocytes (Fig. 7B, C). Then, we sought to explore whether these MSC-EVs-Agomir^miR−181a−5p^ decreased the ER stress-induced apoptotic pathway in cardiomyocytes exposed to Dox. The result showed that MSC-EVs-Agomir^miR−181a−5p^ could partly enhance the suppression toward the entire pathway compared to MSC-EVs-Agomir^NC^ (Fig. 7D, E). Subsequently, we observed the morphological characteristics of cardiomyocytes exposed to Dox and MSC-EVs-Agomir^miR−181a−5p^ or MSC-EVs-Agomir^NC^, which further indicated that MSC-EVs-Agomir^miR−181a−5p^ maintained the normal morphology of cardiomyocytes better (Fig. 7F). Finally, we evaluated the apoptotic proportion of cardiomyocytes exposed to Dox, which revealed that MSC-EVs-Agomir^miR−181a−5p^ had a better antiapoptotic effect on cardiomyocytes exposed to Dox compared to Dox and MSC-EVs-Agomir^NC^ (Fig. 7G). Taken together, the cardioprotective ability is enhanced after increasing miR-181a-5p in MSC-EVs toward Dox-induced cardiomyopathy.

## Discussion

The robust cardioprotective potentials of intravenous MSCs have been widely recognized in clinical trials, while the inability to explain how lung-trapped MSCs affect the injured myocardium impedes its wide application in the cardiovascular field [44, 45]. Furthermore, two ongoing clinical trials (ClinicalTrials.gov Identifier: NCT02408432 and NCT02962661) sponsored by the Anderson Cancer Center of M.D. also aim to investigate the relevant cardioprotective ability of intravenously injected MSC therapy on anthracyclines secondary cardiotoxicity. Therefore, before clinical usage, it is urgent to conduct an animal study to determine potential mechanisms, which makes intravenously infused MSCs be one of the mechanism-oriented therapies.

**Fig. 8:**
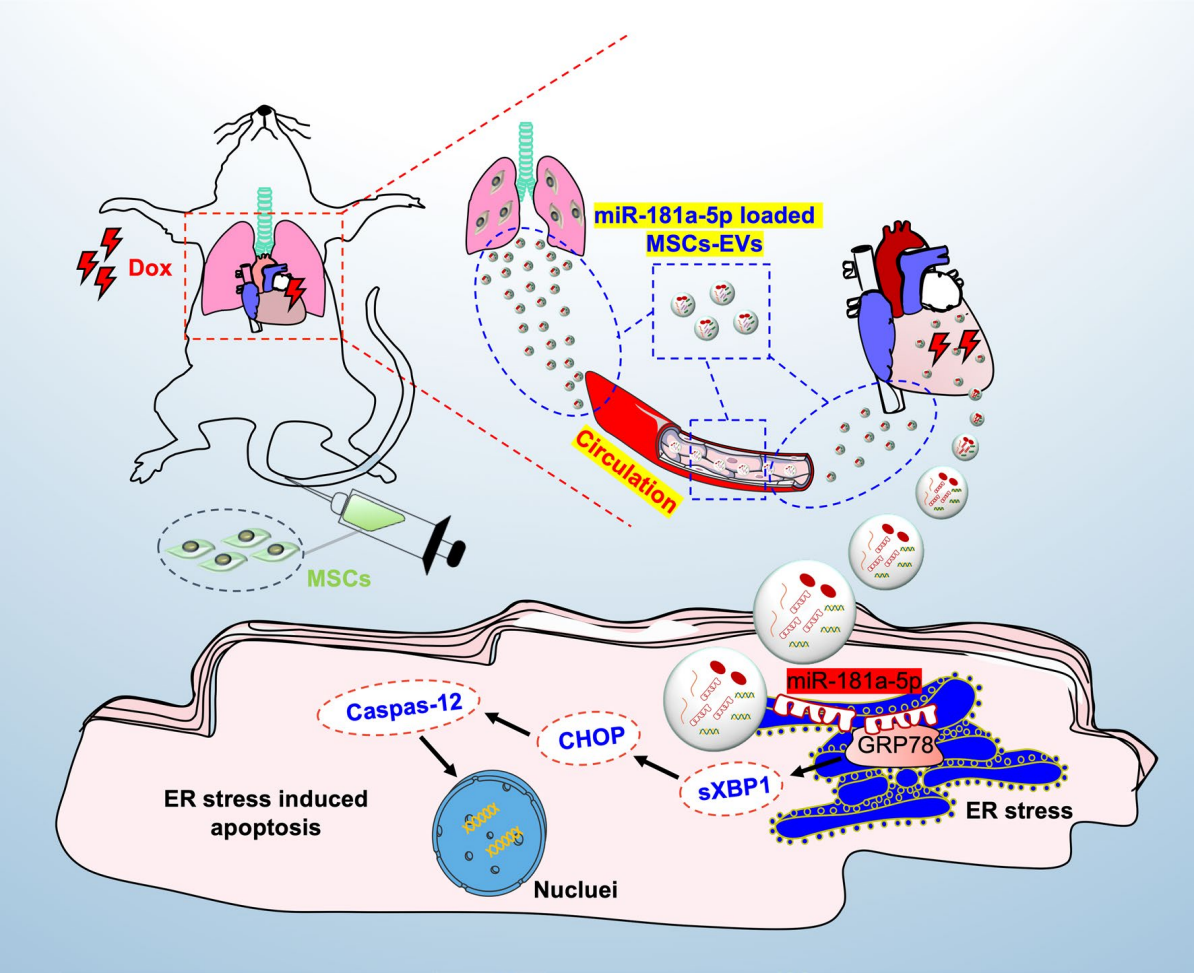
Schematic illustration of the role of intravenously injected MSCs on cardioprotection. Intravenously transplanted mesenchymal stromal cells (MSCs), as an endocrine reservoir, released functional extracellular vesicles (EVs) from the lung to the heart continuously and gradually. Specific component in MSCs-derived EVs once delivered to injured myocardium is identified to alleviate doxorubicin (Dox)-induced cardiotoxicity through suppressing endoplasmic reticulum (ER) stress-induced cardiomyocyte apoptosis

In this study, consecutive doses of MSCs were optimized injecting strategy to alleviate Dox-induced cardiotoxicity through reducing Dox-induced ER stress-induced apoptosis in cardiomyocytes. Moreover, we identified that consecutive doses prolonged dwell time of MSCs in vivo which contributes to continuous and gradual release of MSC-EVs from the lung to myocardium to execute trans-organ communication between MSCs and recipient cardiomyocyte with the vehicles of peripheral blood. For Dox-induced cardiotoxicity, miR-181a-5p might be one of the cardioprotective noncoding RNAs (Fig. 8).

Intravenous infusion of MSCs has been recognized as a promising treatment in cardiovascular diseases including Dox-induced cardiotoxicity, while current evidence failed to clarify how these lung-trapped MSCs affect cardiac repair systematically [46]. The prolonged retention of MSCs in mice contributes to improved therapeutic ability. Motivated by this biological basis, we first investigated the transplanted cell retention in mice. Our BLI validated that the majority of intravenously infused MSCs were trapped and disappeared in lung tissue quickly with undetectable retention in the myocardium [47]. Theoretically, intravenously transplanted MSCs hardly give rise to direct cardiac repair. Furthermore, accumulating studies described that the therapeutic effect of intravenously injected MSCs on cardiovascular diseases is dose-dependent, which implied that consecutive doses are superior to single dose in the treatment [47–49]. Moreover, our study indicated that there was a certain percentage of sudden death after administering a high dose of MSCs, while few infusion-related deaths occurred by low-dose MSCs, which demonstrated that consecutive doses can be a preferable delivery strategy for further clinical practice. These data support that consecutive doses of MSCs are more suitable for treating cardiovascular diseases, which is consistent with the conventional pharmacological concept that repetitive doses facilitate the maintenance of constant plasma drug concentration.

As one of the critically intercellular communication, trans-organ communication is described as cells from a certain organ communicate with recipient cells from distant organs through specific signals by gradual, dynamic, and continuous interchange [11, 50]. Undoubtedly, emerging evidence demonstrates that among endogenous cells, this trans-organ communication plays an essential role in physiological and pathophysiological activity [11, 39, 51, 52]. During this process, peripheral blood plays a critical role in delivering certain signals to confer this communication [51–53]. It is worth noting that these signals are always encapsulated into EVs, which minimize the destruction of certain blood components and consist of serum complexity [39, 50, 54–57]. Previous literature reveals that both adipocytes and vascular endothelial cells confer such trans-organ communication to accelerate the cardiac repair through EVs in the transverse aortic constriction and myocardial ischemia/reperfusion model [12, 50]. For transplanted MSCs, EVs secreted from MSCs could also be released into the blood and delivered into relevant distant recipient cells. Unlike intravenous injection of MSC, the intravenously injected MSC-EVs can be cleared from blood promptly and retained in the liver and spleen [20]. These direct injected MSC-EVs are not capable of redistribution from the liver and spleen to the heart, which highlights the unique superiority of intravenously injected MSCs.

To monitor this trans-organ communication in vivo, we modified two kinds of MSCs genetically by introducing a human ubiquitin promoter driving *Fluc*-eGFP double-fusion gene or Gluc-lactadherin fusion protein. Previous literature from our group reveals that Gluc-MSCs had strong Gluc activity in EVs released by Gluc-MSCs [14]. In addition, genetically modified DF-MSCs release eGFP^+^ EVs, which can be detected using fluorescence-based techniques, and our current results also showed detectable expression of eGFP in EVs derived from DF-MSCs by FACS [58]. Thus, all the techniques contribute to the detection of MSC-EVs from total serum EVs. Through our FACS data, temporal change in eGFP signals was detectable in serum EVs from mice administered with the third dose of MSC intravenously. Additionally, Gluc activity can also be detected in serum EVs collected from mice administered Gluc-MSCs but not MSCs-WT. All data support that intravenously injected MSCs release a certain volume of EVs into the peripheral blood. Regrettably, we were unable to further detect eGFP expression in the myocardium through immunofluorescence staining, which might be attributed to the low eGFP^+^ rate in all MSC-EVs and the wide distribution of MSC-EVs in the myocardium. Therefore, to overcome this limitation, miR-181a-5p, as the true therapeutic effector, was detected in total serum EVs and myocardium.

How EVs achieve their biological functions is always determined by their inner active biomolecules, including mRNAs, miRNA, lipids, and proteins [53, 55]. Due to the double-layer membrane structure, the inner active biomolecules are well protected from degradation by complements and RNase [59]. Additionally, as EVs were secreted from donor cells and released into the blood, the components within EVs have a better ability to confront the complexity of the blood [12, 50]. A previous study did not verify that certain miRNA from MSC-EVs is delivered to the myocardium, though they elucidate that MSCs are capable of releasing EVs into blood [22]. In this study, we further verified the release of EVs from transplanted MSCs into blood with different technologies and identified miR-181a-5p as a therapeutic effector. Through the detection of the expression of miR-181a-5p in the blood and myocardium, we clarify that these miR-181a-5p-enriched MSC-EVs can be delivered to the myocardium through blood.

Multiple mechanisms contribute to cardiotoxicity as patients are exposed to accumulated Dox, but if we focus on the classical pathological characteristics, the distention of ER draws our attention, which is closely relevant to ER stress in accordance with the result of endomyocardial biopsy from the first clinical case report in long-term Dox-administrated patients, which observed significant ER distention in the myocardium [30, 60–62]. With the overdose of Dox accumulating in the myocardium, the misfolded proteins are overloaded and processed in the ER, leading to ER stress and finally to cell apoptosis finally [63–65]. The whole process is initiated by overloaded GRP78, which has been used as a therapeutic target. In our study, we observed that ER stress was overactivated and then induced cardiomyocyte apoptosis, while ER stress was suppressed once consecutive doses of MSCs were administered intravenously, and the apoptotic proportion of cardiomyocytes decreased dramatically. As we all know, the ER stress-induced apoptotic pathway is triggered by upregulated GRP78, which inspired us to investigate how intravenously injected MSCs suppress the upregulation of GRP78 [65].

It is well known that miRNA can inhibit protein expression by targeting relevant mRNA at the post-transcriptional level, and this miRNA-mRNA interaction can be predicted through multiple miRNA prediction datasheets online. Hence, whether there is a certain miRNA with such an ability to inhibit GRP78 expression in MSCs or MSC-EVs draws our attention. As one of the dominant miRNAs in MSCs, miR181a-5p was included from multiple miRNA datasheets, which can inhibit GRP78 with high efficacy through in vitro experiments. Furthermore, miR-181a-5p expression from both serum EVs and myocardium was increased once repeated low-dose MSCs were administered, which implied that miR-181a-5p-enriched MSC-EVs released from intravenously infused MSCs were delivered directly to the injured myocardium. Although intravenously injected MSCs were trapped in the lung for a limited 48–72 h, consecutive doses increased more miR-181a-5p-enriched MSC-EVs into peripheral blood and myocardium, which promotes cardiac repair.

This study is not without limitations. The main limitation of the current study is that our experimental mouse model is not based on tumor-bearing mice. As an antitumor agent, Dox can only be injected to treat all kinds of tumors, while our study was carried out in normal mice, which is not coincident with clinical practice. However, in other words, the pathophysiological disorder and complexity in tumor-bearing mice may lead to a series of interferences especially in terms of peripheral blood components, which is detrimental to investigating the critical role of trans-organ communication.

## Conclusions

In the present study, we systematically described how intravenously transplanted MSCs, as a new endocrine reservoir, repair the injured myocardium through the release of functional EVs in the treatment of Dox-induced cardiotoxicity. The majority of MSCs were trapped in the lung once transplanted into mice intravenously and then released the functional EVs into blood continuously and gradually followed by the significant enrichment in the myocardium. For the doxorubicin-induced cardiotoxicity, miR-181a-5p encapsulated in MSC-EVs played a critical role through suppressing endoplasmic reticulum (ER) stress-induced myocardial apoptosis.

## Supplementary Information

**Additional file 1:** Supplementary Tables and Figures.

## Data Availability

All relevant data supporting the findings of this work are available within the paper, its Additional file 1, and from the corresponding authors upon reasonable request.

## Abbreviations

3’UTR: 3’ Untranslated regions
BCA: Bicinchoninic acid
BLI: Bioluminescence imaging
BNP: Brain natriuretic peptide
COVID-19: Coronavirus disease 2019
DLS: Dynamic light scattering
Dox: Doxorubicin
DPBS: Dulbecco’s phosphate-buffered saline
EF: Ejection fraction
eGFP: Enhanced green fluorescence protein
ER: Endoplasmic reticulum
EVs: Extracellular vesicles
FBS: Fetal bovine serum
Fluc: Firefly luciferase
FS: Fractional shortening
Gluc: *Gaussia* luciferase
GRP78: Glucose-regulated protein 78
H&E: Hematoxylin and eosin
HW/BW: Ratio of heart weight and bodyweight
MSCs: Mesenchymal stromal cell
MSC-EVs: Mesenchymal stromal cells-derived extracellular vesicles
MRC5-EVs: MRC5-derived extracellular vesicles
PBS: Phosphate-buffered saline
PCR: Polymerase chain reaction
PECAM-1: Platelet endothelial cell adhesion molecule 1
PVDF: Polyvinylidene difluoride
TEM: Transmission electron microscope
TnI: Troponin I
TnT: Troponin T
TRITC: Tetramethylrhodamine
