# Supplementary material for: Intravenously transplanted mesenchymal stromal cells: a new endocrine reservoir for cardioprotection
Source: Stem Cell Res Ther. 2022 Jun 17;13:253. doi: 10.1186/s13287-022-02922-z (PMC9204704; doi:10.1186/s13287-022-02922-z)
Supplement: Supplementary file 1 — Additional file 1: Supplementary Tables and Figures. [file 13287_2022_2922_MOESM1_ESM.docx]

**ADDITIONAL FILE 1**

**Supplemental Table**

**Additional file 1: Table S1. Potential GRP78 targeted miRNA profiles from predictions of TargetScan, miRanda, and miRDB**

| **Mouse GRP78** | | |
| --- | --- | --- |
| **TargetScan** | **miRanda** | **miRDB** |
| miRNA181a-5p | miRNA148a | miRNA495-3p |
| miRNA181b-5p | miRNA152 | miRNA12200-5p |
| miRNA181c-5p | miRNA148b | miRNA1192 |
| miRNA181d-5p | miRNA181a-5p | miRNA379-5p |
| miRNA30a-5p | miRNA181b-5p | miRNA1193-5p |
| miRNA30b-5p | miRNA181c-5p | miRNA6953-5p |
| miRNA30c-5p | miRNA181d-5p | miRNA8118 |
| miRNA30e-5p | miRNA150 | miRNA5124 |
| miRNA384b-5p | miRNA384-5p | miRNA12185-3p |
| miRNA199a-5p | miRNA30a-5p | miRNA3058-5p |
| miRNA199b-5p | miRNA30b-5p | miRNA7647-3p |
|  | miRNA30d-5p | miRNA7a-1-3p |
|  | miRNA30e-5p | miRNA199a-5p |
|  | miRNA329 | miRNA199b-5p |
|  | miRNA362-3p | miRNA5619-3p |
|  | miRNA379 | miRNA350-3p |
|  | miRNA495 | miRNA6974-3p |
|  | miRNA1192 | miRNA1970 |
|  | miRNA543 | miRNA1970e-5p |
|  |  | miRNA148b-5p |
|  |  | miRNA148a-5p |
|  |  | miRNA5619-5p |
|  |  | miRNA668-3p |
|  |  | miRNA338-5p |
|  |  | miRNA879-3p |
|  |  | miRNA1199-5p |
|  |  | miRNA16-1-3p |
|  |  | miRNA1969 |
|  |  | miRNA9b-3p |
|  |  | miRNA6414 |
|  |  | miRNA6970 |
|  |  | miRNA325-3p |
|  |  | miRNA1897-3p |
|  |  | miRNA6981-3p |
|  |  | miRNA669k-5p |
|  |  | miRNA7242-5p |
|  |  | miRNA6896-3p |
|  |  | miRNA150-5p |
|  |  | miRNA5127 |
|  |  | miRNA376c-5p |
|  |  | miRNA376b-5p |
|  |  | miRNA181b-5p |
|  |  | miRNA181a-5p |
|  |  | miRNA181c-5p |
|  |  | miRNA181d-5p |
|  |  | miRNA7212-3p |
|  |  | miRNA6942-3p |
|  |  | miRNA674-3p |
|  |  | miRNA1251-5p |
|  |  | miRNA3473f |
|  |  | miRNA7236-5p |
|  |  | miRNA691 |
|  |  | miR7004-3p |

**Additional file 1: Table S2. MiRNA sequences in human and mouse**

|  |  | **miRNA sequences** |
| --- | --- | --- |
| **miRNA181a-5p** | **Human** | AACAUUCAACGCUGUCGGUGAGU |
|  | **Mouse** | AACAUUCAACGCUGUCGGUGAGU |
| **miRNA181b-5p** | **Human** | AACAUUCAUUGCUGUCGGUGGGU |
|  | **Mouse** | AACAUUCAUUGCUGUCGGUGGGUU |
| **miRNA181c-5p** | **Human** | AACAUUCAACCUGUCGGUGAGU |
|  | **Mouse** | AACAUUCAACCUGUCGGUGAGU |
| **miRNA181d-5p** | **Human** | AACAUUCAUUGUUGUCGGUGGGU |
|  | **Mouse** | AACAUUCAUUGUUGUCGGUGGGU |

**Supplemental Figures & Legends**

** Figure S1. Repeated low-dose MSCs prolonged cell retention in the lung.** 5-day consecutive BLI imaging for mice administrated with a single dose of MSCs (5×10^6^ MSCs/dose) or consecutive doses of MSCs (1×10^6^ MSCs/dose). The BLI signal of intravenously administrated MSCs is undetected on day 5 in mice administrated with dose of MSCs but consistently and steadily detected in mice administrated with consecutive doses of MSCs.

** Figure S2. Intravenous transplanted MSCs triggered no extra lung injury.** Scale bar, 100 μm.

** Figure S3.** **Consecutive doses of MSCs attenuated Dox-induced myocardial dilation. A.** Quantitative analysis of the ratio of heart weight and bodyweight (HW/BW) in mice administrated with PBS (Control), Dox/PBS, Dox/Single dose, or Dox/Consecutive doses on day 7 and 14 (n = 5). ^**^*P* < 0.01 vs Control, ^###^*P* < 0.001 vs Dox/PBS, ^&^*P* < 0.05 vs Dox/Single dose. Data were analyzed by one-way ANOVA followed by Bonferroni post hoc test. **B.** Representative H&E staining images of transversal whole ventricular sections in each group on day 14. Scale bar, 1 mm.

** Figure S4. Consecutive doses of MSCs attenuated Dox-induced cardiac fibrosis. A.** Representative Sirius red staining images of ventricular tissue in mice administrated with PBS (Control), Dox/PBS, Dox/Single dose, or Dox/Consecutive doses on day 14. Scale bar, 100 μm. **B.** Statistics of fibrotic area per left ventricle in each group (n = 5). ^****^*P* < 0.0001 vs Control, ^####^*P* < 0.0001 vs Dox/PBS. ^&&&&^*P* < 0.0001 vs Dox/Single dose. Data were analyzed by one-way ANOVA followed by Bonferroni post hoc test.

**
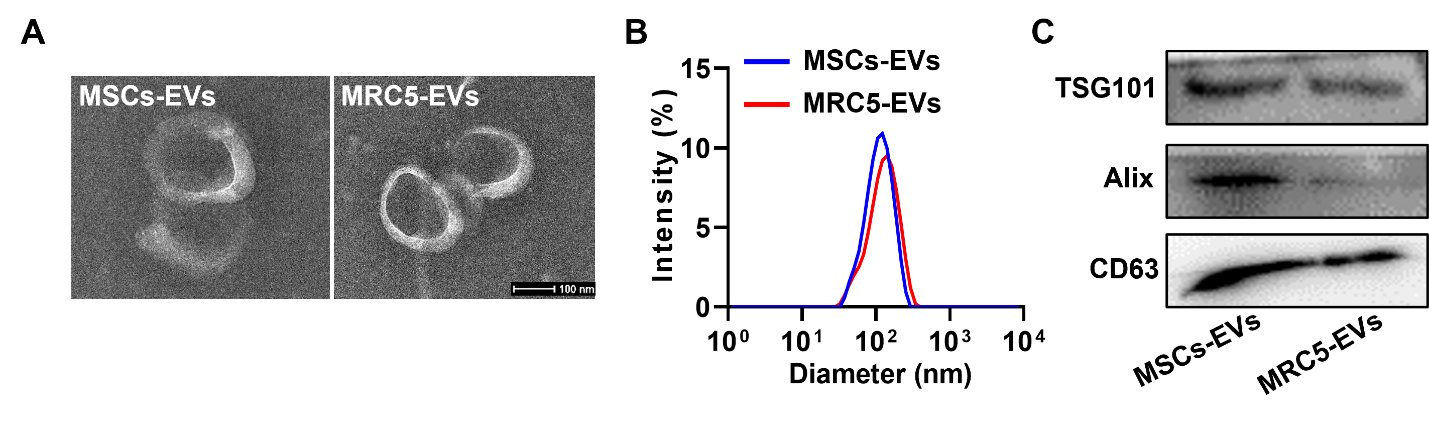
 Figure S5. Characterization of extracellular vesicles (EVs). A.** Representative TEM images of MSC-EVs and MRC5-EVs. Scale bar, 10 nm. **B.** The size distribution of MSCs-EVs and MRC5-EVs. **C.** Representative Western blot images of EVs markers (TSG101, Alix and CD63) expressed on MSCs-EVs and MRC5-EVs.

** Figure S6. Expression of miRNA profiles in MSCs (-EVs) and MRC5 (-EVs). A.** Statistics of relative expression of miR-181a-5p, miR-181b-5p, miR181c-5p and miR-181d-5p in MRC5 and MSCs (n = 5). ^*^*P* < 0.05 vs MRC5. Data were analyzed by unpaired two-tailed Student’s *t-*test. **B.** Statistics of relative expression of miR-181a-5p, miR-181b-5p, miR181c-5p and miR-181d-5p in MRC5-EVs and MSCs-EVs (n = 5). ^**^*P* < 0.01, ^****^*P* < 0.0001 vs MRC5-EVs. Data were analyzed by unpaired two-tailed Student’s *t-*test.

**
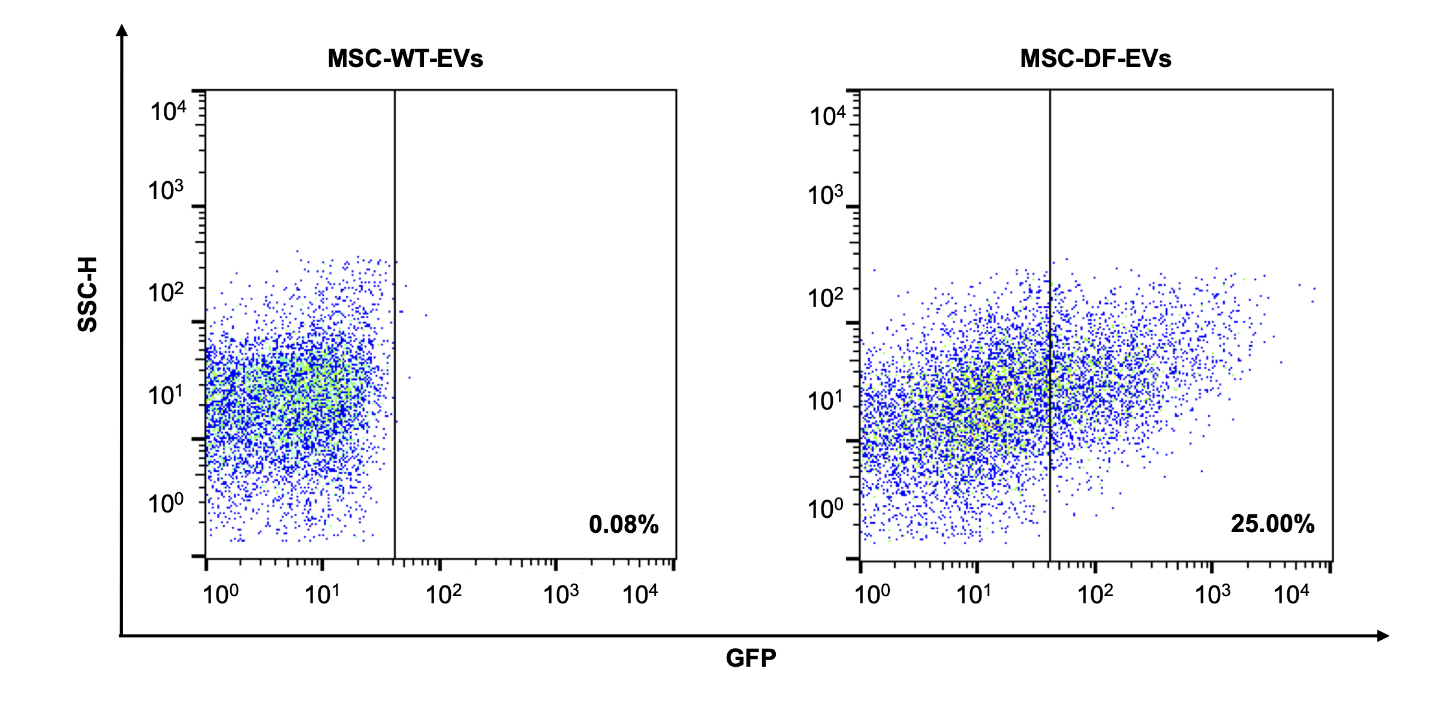
 Figure S7. The quantitative proportion of eGFP^+^ EVs from MSCs-WT and DF-MSCs via FACS analysis.**

**Figure S8. Increased miR-181a-5p levels of serum EVs and lung tissue in mice treated with MSCs. A.** MiR-181a-5p levels of the lung, serum EVs and myocardial tissue in mice injected with PBS or DF-MSCs (n = 3). ^*^*P* < 0.05, ^**^*P* < 0.01 vs PBS. Data were analyzed by one-way ANOVA followed by Bonferroni post hoc test. **B.** MiR-181a-5p levels of liver, spleen, and kidney in mice treated with PBS or MSCs (n = 3).

**Images of the uncropped blots shown in the main figures.**
